# The Fallacy of the Art of Physic, as Taught in the Schools: With the Development of New and Important Principles of Practice

**Published:** 1837-01-01

**Authors:** 


### The Fallacy of the Art of Physic, as taught in the Schools:—with the Development of new and important Principles of Practice. By *Samuel Dickson*, M.D. Ed. 1836.

This volume ought to have been headed—Hahnemania antiquata; or Pyreximania triumphalis. Homœopathy and every other pathy may now hide their heads, for a new system is let loose upon the world which is to eclipse all that preceded it! Hitherto we have been accustomed to deplore the many ills to which flesh is heir. But we were all in error. There is but *one* malady on earth—but, like Goldsmith's fault—it is a "thumper!" "In the sequel we mean to shew that, instead of many maladies, man, *properly speaking*, is the victim only of one." This one disease is fever—not fever in the large sense of the word, but only remittent fever! The process of reasoning by which Dr. Dickson appears to have arrived at this conclusion, is equally short and singular. He considers disease to be simply "an increase or diminution of the proper functions of health"—and as fever answers to this definition—*ergo*, all diseases are remittent fevers! The author of this theory seems to forget that two people may play at the same game. As hydrophobia must be considered as presenting an aberration from health—or to use Dr. D.'s language—an increase or diminution of healthy function—so it might be inferred that all diseases were neither more nor less than hydrophobia! But a part of Dr. D.'s definition of disease is rather curious—an *increase* of healthy function! Thus a man who is rapidly convalescing from typhus fever, and consequently *increasing* in healthy function, must be daily plunging deeper and deeper into disease, according to the definition in question. Indeed we have not, for many years, met with so amusing a book on medicine as the present. Dr. D. has an original way of doing every thing. Thus he expresses the greatest respect and even gratitude to Sir James M'Grigor, the head of the army medical department—as his patron and friend. Now it is well known that Sir James has long impressed upon the army medical officers, the utility—the necessity of cultivating morbid anatomy, and also the employment of the stethoscope. Listen to Dr. Dickson. "That bauble of Laennec—the stethoscope—which the reader (and Sir James) will pardon me for holding in heterodox contempt, is, of course, employed. The very application of the instrument to the chest *deranges the action of the lungs (!!!)* and heart."—16.

After such a passage, demonstrative of such profound ignorance and inveterate prejudice, the reader would probably excuse any farther notice of the work; nor will we trouble him with much more of it. Morbid anatomy, so strenuously urged on the attention of the army medical officers, is quite as much despised by our author as the "bauble of Laennec." The following is a specimen of logic to prove that all diseases are remittent fever, because they all shew *increase of temperature* at one time or other.

"There can be no abnormal action, no local lesion, no change of structure unpreceded by change of temperature. You occasionally, it is true, meet with patients who, from their minds being exclusively wrapt up in the local prominent feature for which they consult you, will deny this with hardihood; but a cross-examination in most instances brings them to stultify their previous denial. In rare cases—cases where the memory is defective, or where the patient is so self-sufficient as to pretend to instruct you—you will fail even in this. It is not, however, the less true on that account.

Take the subject of pulmonary consumption. He labours under mental inquietude, which his position so often places beyond his control; or he has been subjected to a bad atmosphere, or exposed to a draught or to the cold night air. In either case he shivers, fevers; or his feet or hands become by turns colder and hotter than natural; perhaps he sweats; then come the cough, the expectoration, both gradually developed by successive returns of the universal disturbance. Mark the patient well. There are days, nay times of the day, when his cough and expectoration diminish, when his constitution is little if at all disturbed. Bodily and mentally he tells you he is better, and there is an amendment; but another day, and the morbid phenomena recur; the remissions are less and less marked; emaciation is more and more perceptible; the night-sweats become troublesome and frequent; and death, under the routine aggravation of leeches, blisters, and palliatives, sooner or later closes the scene. Is not this the history of *every disorder* where the physician fails to make an impression with the resources of his art? Setting aside the mere expectoration, do you not occasionally witness the whole of the above train of symptoms, the cough, the fever, the wasting, the night-sweats, where after death you cannot detect the slightest structural change in the lungs? What then do you term the disorder? Is it not equally consumption, or rather do not both resolve themselves into cases of *remittent fever*, the one differing from the other simply in the *development of local lesion?*" 18.

Gout he maintains to be a "remittent fever," on the authority of Sydenham, who tells us that the pain remits in about 24 hours from the beginning of the attack! "Cancer, lumbar, hepatic, and other abscesses, auricular, vaginal, and other discharges, the scrofulous, and rheumatic joint, diseased kidney and bladder, and every variety of eruption, ulcer, and humour, including *aneurism*," &c. are all remittent fevers!

If this be madness, "there is method in it"—witness the following passage:—

"If multiplicity of patients be at all a test of successful treatment, the profession may draw a favourable inference from the fact, that in a less period than three years, I have prescribed for upwards of *eight thousand* private patients,—depletion in no instance forming a part of the treatment." 53.

What! no *depletion* of your patient's purses? Dr. Dickson scorns to do things like other people. He disdains to beat about the bush, but launches out into as considerable a "puff direct," as we have ever witnessed in any medical work not the offspring of a professed charlatan!

We have only to add, in conclusion, that such works as these, including Hahnemania, do incalculable injury to the profession by impressing on the minds of the public that we have no steady principles to guide us, but are blown about by every gale, and thrown into sects and parties by every new doctrine, however absurd. It is, perhaps, fortunate that this last absurdity will have a very limited circulation—namely, that it will be confined almost exclusively to the author's own private patients, gratuitously distributed. Still, when we consider that these amount to about *three thousand* per annum, the mischief will be considerable.

---

